# High dose vitamin D_3_ empowers effects of subcutaneous immunotherapy in a grass pollen-driven mouse model of asthma

**DOI:** 10.1038/s41598-020-77947-6

**Published:** 2020-11-30

**Authors:** Laura Hesse, N. van Ieperen, Arjen H. Petersen, J. N. G. Oude Elberink, Antoon J. M. van Oosterhout, Martijn C. Nawijn

**Affiliations:** 1 Department of Pathology and Medical Biology, Experimental Pulmonary and Inflammatory Research (EXPIRE), University Medical Center Groningen (UMCG), Groningen Research Institute of Asthma and COPD (GRIAC), University of Groningen, Internal Postcode EA52, Hanzeplein 1, 9713 GZ Groningen, The Netherlands; 2 Groningen Research Institute of Asthma and COPD (GRIAC), University Medical Center Groningen, University of Groningen, Groningen, The Netherlands; 3 Department of Pathology and Medical Biology, Medical Biology Section, University Medical Center Groningen, University of Groningen, Groningen, The Netherlands; 4 Division of Allergy, Department of Internal Medicine, University Medical Centre Groningen, Groningen, The Netherlands

**Keywords:** Immunization, Immunosuppression, Adaptive immunity, Immune tolerance

## Abstract

Allergen-specific immunotherapy (AIT) has the potential to provide long-term protection against allergic diseases. However, efficacy of AIT is suboptimal, while application of high doses allergen has safety concerns. The use of adjuvants, like 1,25(OH)_2_VitD_3_ (VitD3), can improve efficacy of AIT. We have previously shown that low dose VitD3 can enhance suppression of airway inflammation, but not airway hyperresponsiveness in a grass pollen (GP)-subcutaneous immunotherapy (SCIT) mouse model of allergic asthma. We here aim to determine the optimal dose and formulation of VitD3 for the GP SCIT. GP-sensitized BALBc/ByJ mice received three SCIT injections of VitD3-GP (30, 100, and 300 ng or placebo). Separately, synthetic lipids, SAINT, was added to the VitD3-GP-SCIT formulation (300 nmol) and control groups. Subsequently, mice were challenged with intranasal GP, and airway hyperresponsiveness, GP-specific IgE, -IgG1, and -IgG2a, ear-swelling responses (ESR), eosinophils in broncho-alveolar lavage fluid and lung were measured. VitD3 supplementation of GP-SCIT dose-dependently induced significantly enhanced suppression of spIgE, inflammation and hyperresponsiveness, while neutralizing capacity was improved and ESR were reduced. Addition of VitD3 further decreased Th2 cytokine responses and innate cytokines to allergens in lung tissue by GP-SCIT. However, addition of synthetic lipids to the allergen/VitD3 mixes had no additional effect on VitD3-GP-SCIT. We find a clear, dose dependent effect of VitD3 on GP-SCIT-mediated suppression of allergic inflammation and airway hyperresponsiveness. In contrast, addition of synthetic lipids to the allergen/VitD3 mix had no therapeutic effect. These studies underscore the relevance of VitD3 as an adjuvant to improve clinical efficacy of SCIT treatment regimens.

## Introduction

Allergen-specific immunotherapy (AIT) is a treatment for allergic disorders that has the potential to induce long-term relieve from allergic symptoms and an immunological state of tolerance towards allergens. Successful AIT leads to reduced use of medication and improved quality of life^1,2^. AIT is a unique form of therapy wherein allergens are administered via subcutaneous injection (SCIT) or sublingual application (SLIT)^3,4^. AIT has been shown to result in increased regulatory T cell numbers and activity, and suppression of numbers and activity of allergen-specific Th2 cells and type 2 innate lymphoid cells (ILC2s) and concomitant inhibition of eosinophilic inflammation. In addition, AIT induces a neutralizing antibody response that results in mast cell and basophil desensitization^3,5^. Despite the ability of AIT to induce long term tolerance to allergens, AIT is not a routine treatment for allergic asthma due to variable efficacy and long treatment duration. Moreover, the use of crude allergen extracts with IgE-crosslinking capacity has safety concerns^6^. Strategies to improve AIT regimens include alternative administration routes to achieve optimal antigen presentation at low levels of applied allergens^7,8^, use of purified or recombinant allergens and or peptides^9,10^, or addition of an adjuvant to enhance tolerance induction^11^.

Use of adjuvants in AIT aims to increase allergen delivery to and uptake by antigen presenting dendritic cells (DCs) and to enhance their tolerogenic capacity. One candidate adjuvant for AIT, 1,25-dihydroxy-vitamin D3 (VitD3), binds to its nuclear hormone VitD3 receptor, and provides immunoregulatory properties through induction of tolerogenic DCs^12^. VitD3 prevents DC-maturation leading to down-regulation of costimulatory molecules (CD40, CD80, CD86) and enhanced IL-10 production^13^, facilitating the generation of adaptive Treg cells^14^. We have previously shown the successful use of VitD3 supplementation in AIT in the classical mouse model of ovalbumin-induced allergic airway inflammation^15^ as well as in experimental SCIT and SLIT models of grass pollen (GP) AIT^11^. Moreover, VitD3 supplementation in SCIT using house dust mite (HDM) extracts in a clinical study had (modest) positive effects on asthma symptom scores compared to control HDM-SCIT treatment^16^.

Although the urgent clinical need for optimized vaccine formulations, few studies to date have been exploring the beneficial use of liposomes in AIT^17^. Lipid bilayers encapsulating allergens form liposomes, like nanoparticles and act as delivery vehicle, form a depot or function as an adjuvant. One RDBPC trial in patients with allergic asthma treated with liposome-encapsulated HDM-extract showed promising results in inducing blocking IgG responses and lowered eosinophil numbers, although no safety data were reported in this study^18^. In line herewith, a murine model of HDM allergy was used to test intranasal application of liposome-adhered major allergens (*Dermatophagoides pteronyssinus, Der p*) Der p1 and Der p2, and found to be equally effective in lowering Th2 responses but proved superior in increasing Treg cytokines, like IL-10 and TGF-β, when compared to the crude extract alone^19^.

Due to the lipophilic nature of VitD3, simultaneous administration of both poorly water-soluble VitD3 and a freeze dried highly water-soluble GP-extract in an AIT mixture is not optimal. Notwithstanding, simultaneous delivery of VitD3 and the allergen extract in a single injection is desirable in order to tolerize the DCs presenting the allergens, without otherwise influencing the host immune response. Optimizing a formulation of the mixture of the hydrophilic allergen extract and the lipophilic VitD3 might enhance therapeutic efficacy. SAINT-18 (1-methyl-4-(cis-9-dioleyl)methyl-pyridinium-chlorid, SAINT) is a synthetic bio-compatible lipid which can form liposomal structures, and might be able to act as a carrier for VitD3 while encapsulating the GP extract^20^. We therefore hypothesize that use of SAINT in GP-SCIT would enhance tolerance induction after GP-SCIT injections.

Previously, we showed that VitD3 supplementation in GP-SCIT augments induction of neutralizing antibody responses, and leads to enhanced suppression of eosinophilic inflammation and production of IL-10 in lung tissue. Therein, we used a single low dose of 10 ng VitD3, which did not augment suppression of airway hyperresponsiveness (AHR) by GP-SCIT, indicating that this low dose was not optimal^11^. In the current novel study, we aim to find the optimal VitD3 dose in our GP-SCIT model for optimal suppression of all parameters of allergic airway disease. Here, we show for the first time that high dose VitD3 is in fact capable of reducing AHR, Th2 cytokine production and numbers of eosinophils in the lung. In addition, we aim to test whether use of the synthetic lipid SAINT in the mix of GP-SCIT extracts and VitD3 enhances suppression of parameters of allergic inflammation in an experimental mouse model of GP-SCIT. Herein, we clearly show a reproducible beneficial effect of VitD3 on the efficacy of GP-SCIT.

## Materials and methods

### Animals

BALB/cByJ mice (8–9 weeks-old) were purchased from Charles River (L’Arbresle, France) and bred in individually ventilated cages and fed a hypo-allergen GP-free diet (4 kcal/g, 25% protein, 11% fat, 47% sugars, 5% fibers; AB Diets, Woerden, The Netherlands), which has a theoretical pre-manufacture level of 2900 IU/kg Vitamin D3. Due to the high sensitivity of vitamin D3 to light, air, heat and humidity, the actual level of Vitamin D3 might alter during manufacture. Female 7–9-week-old progeny were used for experiments (N = 8). The Institutional Animal Care and Use Committee (DEC) at the University of Groningen approved experiments under license number DEC6209 and all experiments were performed in accordance with relevant guidelines and regulations. Similar experimental and materials descriptions can be found in previous experiments^21^.

### Induction of allergic asthma and treatment protocols

**Figure 1:**
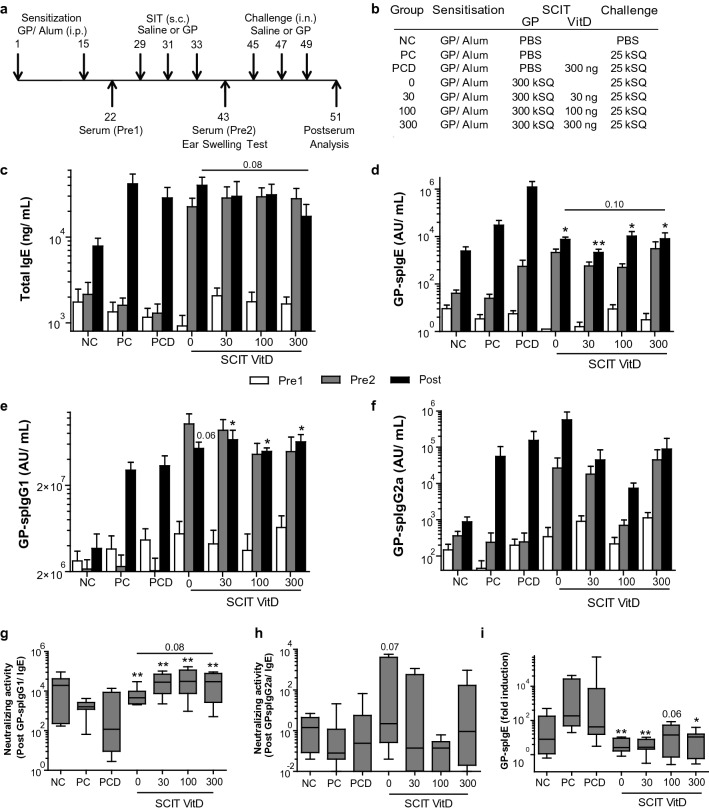
Overview and immunoglobulin response after VitD3-supplemented GP-SCIT. (**a**) Experimental protocol. (**b**) Treatment groups. (**c**) Serum total IgE (ng/mL) before SCIT (white, Pre1), before challenge (grey, Pre2), and after challenge (black, Post). (**d**) Serum GP-spIgE (Arbitrary Units (AU)/mL). (**e**) GP-spIgG1. (**f**) GP-spIgG2a. (**g**) Neutralizing activity plotted as ratio of GP-spIgG1/GP-spIgE in Post-sera. (**h**) GP-spIgG2a/GP-spIgE. (**i**) Fold induction of GP-spIgE (Post/Pre2). (**c–f**) mean ± SEM (n = 8). (**g**–**i**) Box-and-whiskers (min–max). *NC* negative control; *PC* positive control; *PCD* PC with VitD3; 0, 30, 100, and 300 groups all contain GP with 0, 30, 100, and 300 ng VitD3 respectively. *P < 0.05, **P < 0.01, ***P < 0.001 compared to PC or PCD: unless otherwise specified.

On experimental days 1 and 15, all mice received two intraperitoneal injections of 5000 standardized quality (SQ) units (5kSQ = 8 μg allergen extract of GP (*Phleum pratense*, Phl p; ALK-Abelló, Hørsholm, Denmark) adsorbed to 1.6 mg Alum (Imject Alum, Pierce, USA) in 100 µL Phosphate-buffered Saline (PBS, Fig. 1a,b and 4a,b).

Two weeks thereafter, SCIT was performed by three 100 µL s.c. injections, containing either saline or GP with or without 1α,25-dihydroxyvitaminD3 (VitD3, Sigma-Aldrich, The Netherlands). In a separate experiment, new freshly prepared formulations were made containing GP, VitD3 and or SAINT-18. In short, 300 nmol SAINT-18 (stock in WFI, Water for Injection) was mixed with 300 ng VitD3 (stock in ethanol) and then 300kSQ GP was added all in glass vials. Of all formulations, the particle size was established using the Nanotrac Flex In-situ Analyzer (Microtrac, Germany) and considered suitable for injection.

Inhalation challenges were administered as droplets of 25kSQ GP in 25 μL PBS after light isoflurane anesthesia on days 45, 47 and 49. After 2 days, airway responsiveness was determined, and serum samples, broncho-alveolar lavage fluid (BALF), and lung lobes were stored for further analyses (− 80 °C). Both experiments were performed using different batches of rough extract grass pollen (*Phleum pratense*, 225; ID nr. 1031225. Batch numbers: Phl0035 and Phl0043 for research only)^10,22^.

### Early phase hypersensitivity: EST

After SCIT, an ear-swelling test (EST) was performed to evaluate the early phase response to GP. Herein, 10 μL of PBS is injected intradermal in the left ear as a control and 1kSQ of GP in 10 μL is injected in the right ear of mice under isoflurane/oxygen anesthesia. After 2 h, ear thickness was measured using a digimatic force-micrometer at 0.5 N (± 0.15 N, Mitutoyo, Japan) and the net GP-induced swelling (Δ, in mm) was calculated by subtracting the thickness of the left ear from the right ear^22,23^.

### Airway responsiveness

By measuring airway resistance (R in cmH_2_O s/mL) in response to intravenous administration of increasing doses of methacholine (Sigma-Aldrich) the airway responsiveness was assessed. Next, lung compliance (C in mL/H_2_O) was examined as a measure of the comparative stiffness of the lung. In short, anesthetized mice were tracheotomized, cannulated through the jugular vein, and attached to a small animal ventilator; the FlexiVent (SCIREQ, Canada), and ventilated (280 breaths/min) with a tidal volume of 10 mL/kg, pressure limited at 300 mmH_2_O. In response to increasing dosages of methacholine, the airway resistance was calculated from the pressure response to a 2-s pseudorandom pressure wave. In analyzing the peak resistance (R) and peak compliance (C), all values with a coefficient of determination (COD)-value below 0.85 were excluded. Moreover, responsiveness was expressed as the effective dose (ED) of methacholine required to induce a resistance of 3 cmH_2_O s/mL (ED_3_)^9,22^.

### Evaluating inflammation in BALF

Similar as in previous methods descriptions^21^, lungs were lavaged with 1 mL PBS containing 5% Bovine Serum Albumin (BSA, Sigma Aldrich, Zwijndrecht, The Netherlands) and a cocktail of protease inhibitors (Complete mini tablet; Roche, Germany), directly after AHR measurements. Subsequently, four lavages were performed with 1 mL non-supplemented PBS. After centrifugation (500 × *g*, 4 min), the cell-free supernatant of the first mL was stored as BALF (in duplo, − 80 °C). The cells from the first mL were added to the cells from the 4 mL PBS lavages and counted using the Z2 coulter particle count and size analyzer (Beckman Coulter, Woerden, The Netherlands). Cytospin preparations of the BALF and Lung cells were stained with Diff-Quick (Merz&Dade, Dudingen, Switzerland) and 300 cells per cytospin were evaluated and differentiated into mononuclear cells (M), neutrophils (N), and eosinophils (E) by standard morphology.

### Analysis of T cell responses: lung cell-restimulation

As described before^21^, the left lobes of the lungs were removed, minced and digested for 1.5 h at 37 °C in 2 mL of RPMI1640 (Lonza, Breda, The Netherlands) containing 1% Bovine Serum Albumin (BSA), 4 mg/mL collagenase-A (Roche Diagnostics, Almere, The Netherlands) and 0.1 mg/mL DNAse-I (Roche Diagnostics). Next, lung cells were forced though a 70 μm cell strainer (Falcon, Lelystad, The Netherlands), suspended in lysis buffer, washed and suspended again in 10 mL RPMI1640 containing 1% BSA. Total cell counts were established using the Beckman Coulter Counter Z2. Lung cells (5 × 10^5^ cells/well) were cultured in RPMI1640 supplemented with 5% Fetal Calf Serum (FCS, Lonza, Breda, The Netherlands), 2 nM Ultra-GlutaMAX (Life Technologies, Bleiswijk, The Netherlands), 100EU penicillin, and 100 μg/mL streptomycin in a 96-wells plate (Greiner BioOne, Hannover, Germany) in the presence of 0 μg or 30 μg of GP per well. All samples were stimulated in triplicate for 120 h (CO_2_ incubator, 37 °C) and the supernatant was collected and stored (− 80 °C). ELISA determined concentrations of IL-5, IL-10 and IL-13, according to the manufacturer’s instructions (BD Pharmingen, San Diego, CA). The lower detection limits of the ELISAs were 32 pg/mL for IL-5, 10 mg/mL for IL-10, and 15 pg/mL for IL-13.

### GP-specific immunoglobulins

Similar experimental and materials descriptions can be found in previous experiments^21^. Briefly, blood was collected in MiniCollect serum tubes (Greiner Bio-One, Alphen a/d Rijn, The Netherlands) at several time points via orbital puncture (pre-sera) and after the experiment via the vena cava inferior (post-sera, Figs. 1a and 4a). Briefly, for GP-spIgE, NUNC MaxiSorp flat-bottom 96-well plates (Sigma, MO) were coated with 1 μg/mL anti-mouse IgE (BD Pharmingen) in PBS (overnight, 4 °C), washed five times (wash buffer; PBS 0.05% Tween-20), blocked using 3% skimmed milk powder (ELK, Campina, Amersfoort, The Netherlands) in ELISA buffer, and sera samples (diluted 1:8 in PBS 1%BSA) were incubated for 2 h (room temperature). Then, the plates were incubated with 100 μL 1:100 biotin labeled GP in PBS 1%BSA (homemade) for 1.5 h, washed five times and incubated for 1 h with Streptavidin–Horseradish Peroxidase (1:200, R&D Sytems). Again plates were washed, following which SigmaFast™ OPD substrate (Sigma-Aldrich) was added and incubated for 8 min. The reaction was stopped by adding 75 μL of 1.8 M H_2_SO_4_. Optical density (OD) values were measured at 490 nm and analyzed using a classic logit-log transformation model.

For GP-spIgG1 and GP-spIgG2a, plates were coated using 10 μg/mL rough extract GP, blocked using 3%BSA in ELISA buffer, incubated with sera samples (1:300,000 for GP-spIgG1 and 1:100 for GP-spIgG2a), and labeled using biotinylated anti-mouse IgG1 or -IgG2a (1 μg/mL, BD Pharmingen). Concentrations were calculated according to the standard curve (using reference serum) and the results are expressed as arbitrary unit (AU)/mL.

Biotinylation of GP extract was performed using EZ-link Sulfo-NHS-LC-Biotin according to the manufacturers operating instructions (Thermo Scientific) and using a Slide-A-Lyzer cassette (3.5 K MWCO, Thermo scientific) for purification by dialysis overnight^23^.

### Cytokine levels in lung tissue

The right superior lung lobe was used for measurement of total protein content and a cytokine profile. First, lungs were weighed, homogenized and dissolved in Luminex buffer (weight to volume ratio 1:5) and the total protein content was measured using a BCA protein assay according to manufacturer’s protocol (Thermo Scientific, USA). Concentrations of IL1α, IL-4, IL-5, IL-10, IL-13, IL-17, IL-33, IFNγ, Eotaxin (CCL11), GM-CSF, KC and MIP3α (CCL20) were measured using a multiplex Mouse Cytokine/Chemokine Magnetic Bead Panels (MILLIPLEX Map Kit; Merck Millipore, Germany) according to manufacturer’s protocol. Plates were analyzed using a MAGPX1023 4002 with Luminex xMAP technology. Parts of these methods descriptions can also be found in a previous publication by Hesse et al.^21^.

### Statistical analysis

All data are expressed as mean ± SEM. The Mann–Whitney *U* Test was used to analyze the results, and *p* < 0.05 was considered significant. Within the ELISA data, an AU-value which was more than three times the interquartile (IQ) range higher than the upper Q or more than three times the IQ range lower than the lower Q was considered to be an extreme outlier and was removed for further analysis. Within the AHR measurements, to compare the entire curve between groups, a generalized estimated equation (GEE) analysis was used, using SPSS Statistics 20.0.0.2^24^.

## Results

### High-dose VitD3 decreases spIgE and induces neutralizing capacity

We have previously shown that addition of 10 ng VitD3 per injection enhanced suppression of airway inflammation by GP-SCIT^11,23^. Here, we aimed to determine the optimal dose of VitD3 supplementation in GP-SCIT needed to achieve suppression of both airway inflammation and AHR upon GP challenges in sensitized mice. To this end, we tested a dose range of VitD3 (0, 30, 100, 300 ng) in our established model for GP-SCIT^23^ (Fig. 1a,b). To examine whether VitD3 supplementation changed the GP-SCIT-induced immunoglobulins, we measured total IgE, GP-spIgE, GP-spIgG1, and GP-spIgG2a in sera taken before SCIT (white, Pre1), before challenges (grey, Pre2), and after challenges (black, Post, Fig. 1c–f). GP-SCIT induced increased levels of total and GP-specific IgE, as well as increased levels of spIgG1 and sp-IgG2a in all experimental groups. After subsequent challenges, total and spIgE responses were blunted in all GP-SCIT groups compared to sham-treated controls (Fig. 1c,d). Although addition of VitD3 to GP-SCIT did not enhance spIgG2a levels, VitD3 supplementation did result in significantly increased spIgG1 levels at all three dosages used (Fig. 1e,f).

To estimate the allergen-neutralizing capacity, the ratios of GP-spIgG1/GP-spIgE and GP-spIgG2a/GP-spIgE after GP-SCIT were calculated. We find significantly increased ratios in all SCIT groups when compared to control-treated mice (Fig. 1g,h). Moreover, addition of 300 ng VitD3 resulted in a trend towards increased neutralizing capacity when compared to the unsupplemented GP-SCIT group. Finally, the fold increase of GP-spIgE levels induced by allergen challenges (GP-spIgE Post/Pre2) was significantly reduced in all GP-SCIT groups, regardless of the VitD3 dose (Fig. 1i).

### VitD3 supplementation enhances suppression of ear swelling and AHR

**Figure 2:**
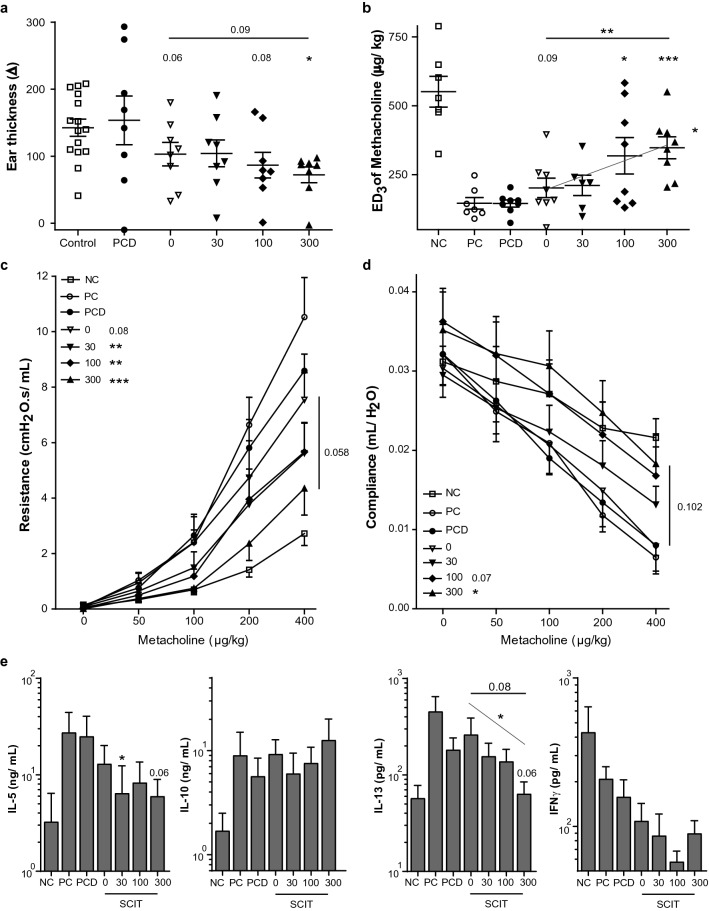
Clinical manifestations after VitD3-supplemented GP-SCIT. (**a**) Net ear thickness (mm) two hours after GP injection performed after SCIT. Placebo-SCIT treated mice were plotted together as Controls (NC and PC). (**b**) Effective Dose (ED) of Methacholine, when the resistance reaches 3 cmH_2_O s/mL (non-parametric Spearman’s rho p < 0.05). (**c**) Airway Resistance (R in cmH_2_O s/mL) and as (**d**) Airway Compliance (C in mL/ cmH_2_O). (**e**) Net levels of IL-5, IL-10, IL-13 (non-parametric Spearman’s rho p < 0.05), and IFNγ measured in restimulated lung single cell suspensions. Absolute values are expressed as mean ± SEM (n = 8). *NC* negative control, PBS challenged; *PC* positive control, GP challenged; *PCD* PC with VitD3 in SCIT (300 ng); 0, 30, 100, and 300 groups all contain 300kSQ GP with 0, 30, 100, and 300 ng VitD3 respectively. *P < 0.05, **P < 0.01, ***P < 0.001 compared to PC or PCD: unless otherwise specified.

We performed an ear swelling test (EST) after GP-SCIT, to evaluate the effects of VitD3 in GP-SCIT on the early-phase allergic response in vivo. High-dose VitD3-GP-SCIT did induce significant suppression of swelling as compared to the VitD3-treated positive controls, and showed a trend for suppression when compared to GP-SCIT without VitD3 (Fig. 2a).

To test whether addition of VitD3 could also enhance GP-SCIT mediated suppression of AHR, we calculated the dose of methacholine required to induce a resistance of 3 cmH_2_O s/mL (ED3; Fig. 2b). Importantly, we observed a clear dose-dependent effect of VitD3 on ED3 after GP-SCIT, indicating that increased VitD3 levels correlated with less severe airway contraction after allergen challenge (Spearman’s rho = *P* < 0.05). Moreover, high-dose VitD3 resulted in a significant increase in ED3 when compared to GP-SCIT without VitD3.

In agreement herewith, airway resistance across the dose–response curve revealed suppression of AHR by all GP-SCIT groups as compared to their matched controls (Fig. 2c). The use of VitD3 in GP-SCIT resulted in a trend towards enhanced suppression compared to control GP-SCIT. Moreover, lung compliance was increased in VitD3-GP-SCIT as compared to the VitD3 supplemented controls and showed a trend towards an increase when compared to the unsupplemented GP-SCIT group (Fig. 2d).

To detect any effect of treatment on allergen-induced cytokines, we assessed cytokine levels in lung cell suspensions restimulated ex vivo with GP extract (Fig. 2e). Here, we observed that VitD3-GP-SCIT mice had reduced IL-5 production after ex vivo GP-stimulation. Moreover, we observed a VitD3 dose-dependent suppression of IL-13 levels after grass pollen restimulation of lung cell suspensions (Spearman’s rho = *P* < 0.05), which resulted in a trend towards reduced IL-13 levels at the highest dose of VitD3 compared to GP-SCIT in the absence of VitD3. Restimulation of lung cell suspensions was unable to alter levels of IL-10 and IFN-γ (Fig. 2e).

### VitD3 enhances suppression of eosinophilic inflammation

**Figure 3:**
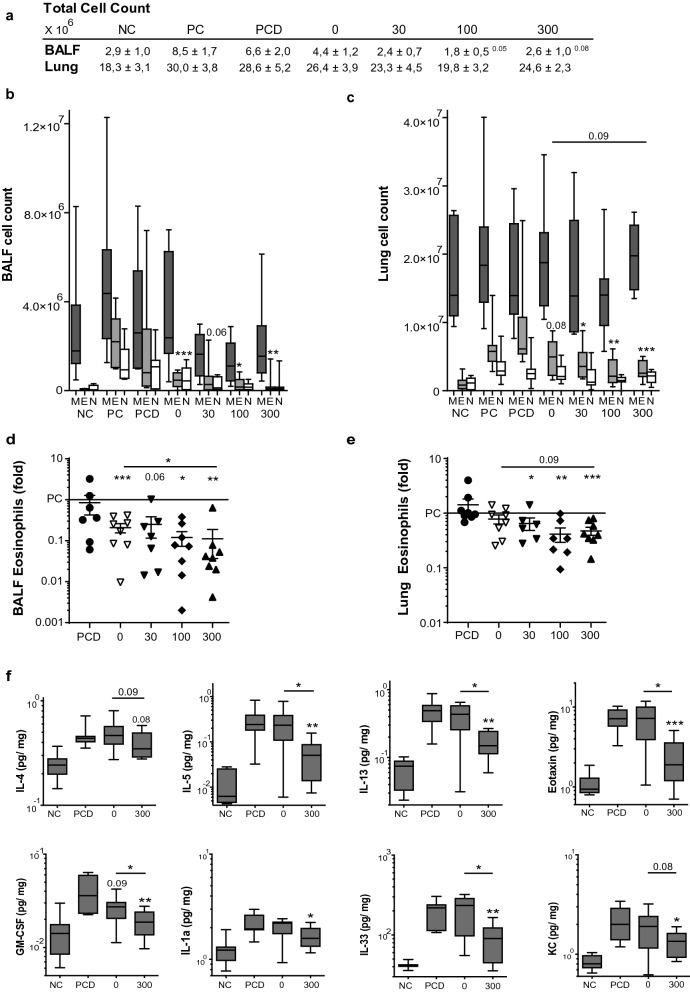
The eosinophilic and cytokine response after VitD3-GP-SCIT. (**a**) Total cell counts in bronchoalveolar fluid (BALF) and lung single cell suspensions (Lung). (**b**) Differential cytospin cell counts in BALF and in (**c**) Lung. *M* mononuclear cells, *E* eosinophils, *N* Neutrophils. Box-and-whiskers plots (min–max). (**d**) BALF and lung eosinophils, both plotted as ratio of suppression (absolute EO/average PC EO; mean ± SEM). **e** Levels of IL-4, IL-5, IL-13, and eotaxin, GM-CSF, IL-1α, IL-33, and KC in pg/mg protein in lung tissue (mean ± SEM, n = 8). *NC* negative control, PBS challenged; *PC* positive control, GP challenged; *PCD* PC with VitD3 in SCIT (300 ng); 0, 30, 100, and 300 groups all contain 300kSQ GP with 0, 30, 100, and 300 ng VitD3 respectively. *P < 0.05, **P < 0.01, ***P < 0.001 compared to PC or PCD: unless otherwise specified.

To evaluate suppression of airway inflammation, we compared cell counts of eosinophilic granulocytes and cytokine levels in lung (Fig. 3a–e). As expected, we found that the number of eosinophils in both BALF and lung tissue is reduced after GP-SCIT (Fig. 3c,d). When assessing VitD3 effects on GP-SCIT mediated eosinophil suppression, we only observed a trend towards enhanced suppression in lung tissue at the highest VitD3 dose as compared to GP-SCIT. To compare the effect of VitD3 in GP-SCIT corrected for any possible baseline effect of VitD3 on eosinophil numbers in lung, we calculated fold reduction in eosinophils of both GP-SCIT groups relative to their respective controls. Here, we observed an enhanced suppression in eosinophil numbers in BALF and lung tissue by GP-SCIT after high-dose VitD3 supplementation (Fig. 3d,e).

Next, we analyzed cytokine levels in lung homogenates and observed that GP-SCIT failed to suppress levels of IL-4, IL-5 and IL-13 in lung tissue in the absence of VitD3. In contrast, levels of IL-4, IL-5 and IL-13 were suppressed by VitD3-GP-SCIT, both compared to the sham-treated and to the GP-SCIT groups (Fig. 3f). To test whether VitD3-GP-SCIT also affected the innate response to allergens, we assessed levels of pro-inflammatory chemokines and alarmins released by lung structural cells upon GP challenges. Interestingly, VitD3 in GP-SCIT resulted in reduced levels of eotaxin (CCL11), GM-CSF, IL-33, and KC when compared to GP-SCIT. Although not significant, similar results were found in IL-1α levels. No significant effects were found in levels of IL-10, MIP3α (CCL20), IFN-γ, and IL-17 (Suppl Fig. 1).

### Unaltered immunoglobulin responses after SAINT complexed to VitD3-GP-SCIT

**Figure 4:**
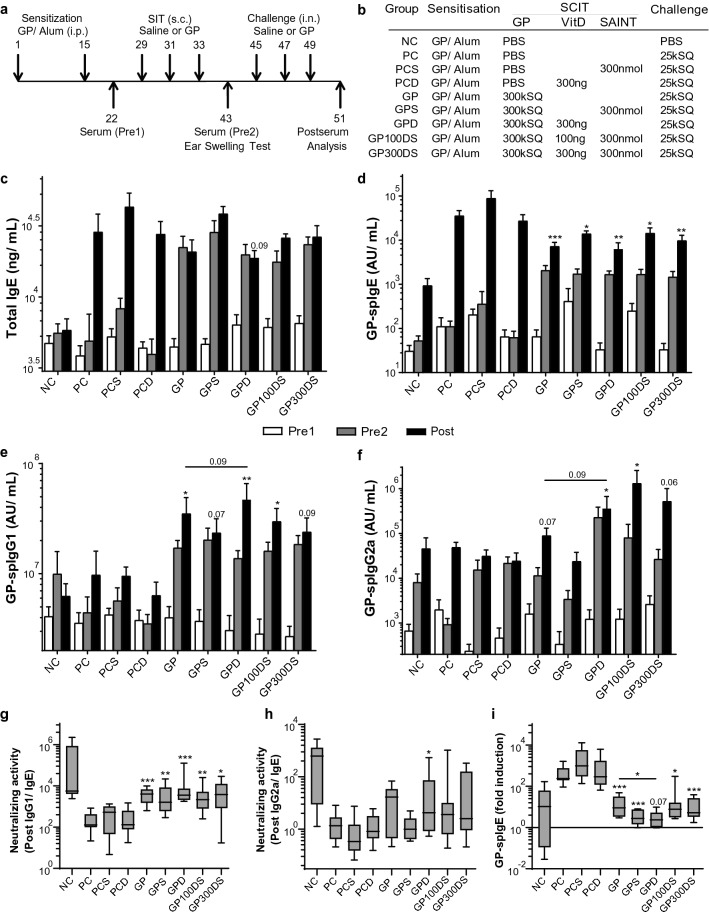
Overview and immunoglobulin response after VitD3-supplemented GP-SCIT. (**a**) Experimental protocol. (**b**) Treatment groups. (**c**) Serum total IgE (ng/mL) taken before SCIT (white bars, Pre1), after SCIT (grey bars, Pre2), and after challenges (black bars, Post). (**d**) GP-spIgE (Arbitrary Units (AU)/mL, Pre1-2, Post). (**e**) GP-spIgG1 (AU/mL, Pre1-2, Post). (**f**) GP-spIgG2a (AU/mL, Pre1-2, Post). (**g**) Neutralizing activity plotted as ratio of GP-spIgG1/GP-spIgE in Post sera. (**h**) Neutralizing activity of GP-spIgG2a/GP-spIgE. (**i**) Fold induction of GP-spIgE after challenge (Post-sera/Pre2-sera). (**c**–**f**) mean ± SEM (n = 8). (**g**–**i**) Box-and-whiskers plots (min–max). *NC* negative control, PBS challenged; *PC* positive control, GP challenged; *PCS* PC with 300 nmol SAINT; *PCD* PC with 300 ng VitD3 in SCIT; *GP* 300kSQ GP in SCIT; *GPS* 300kSQ + 300 nmol SAINT; *GPD* 300kSQ + 300 ng VitD3; *GP100DS* 300kSQ + 100 ng VitD3 + 300 nmol SAINT; *GP300DS* 300kSQ + 300 ng VitD3 + 300 nmol SAINT. *P < 0.05, **P < 0.01, ***P < 0.001 compared to their own matching controls, unless otherwise specified.

Next, we analyzed whether the use of the synthetic lipid SAINT as a carrier for the lipophilic VitD3 in the GP-SCIT formulation could further improve the efficacy of GP-SCIT and the adjuvant effect of VitD3 (Fig. 4a,b). As in the previous experiments, we observed a marked increase in total and GP-spIgE and in spIgG1 and spIgG2a after GP-SCIT (Pre2, Fig. 4c–f), while GP-spIgE responses were blunted after challenges in all SCIT groups compared to their controls (Post, Fig. 4d). However, no differences were found in the spIgE levels obtained from SAINT-VitD3-GP-SCIT groups (GP100DS and GP300DS) when compared to the GP-SCIT groups.

Next, addition of VitD3 to GP-SCIT did show a trend towards increased spIgG1 and spIgG2a, when compared to unsupplemented GP-SCIT (Fig. 4e,f). Although all GP-SCIT groups showed a marked increase in levels of neutralizing antibodies, SAINT as carrier did not result in higher spIgG1 or spIgG2a levels compared to VitD3-GP-SCIT.

As a measure of neutralizing capacity after GP-SCIT, the ratios of GP-spIgG1/GP-spIgE and GP-spIgG2a/GP-spIgE after GP-SCIT were calculated. No effect of VitD3 was observed in these analyses, either with or without use of SAINT as a carrier, although strong induction of neutralizing capacity of spIgG1 was seen after GP challenges in all SCIT groups (Fig. 4g,h). Furthermore, we observed a striking decrease in fold induction of GP-spIgE levels by allergen challenges in all GP-SCIT groups, with a significant decrease in the VitD3 supplemented GP-SCIT treated group when compared to GP-SCIT alone (Fig. 4i).

### SAINT mixed VitD3-GP-SCIT does not enhance suppression of AHR or ear swelling

**Figure 5:**
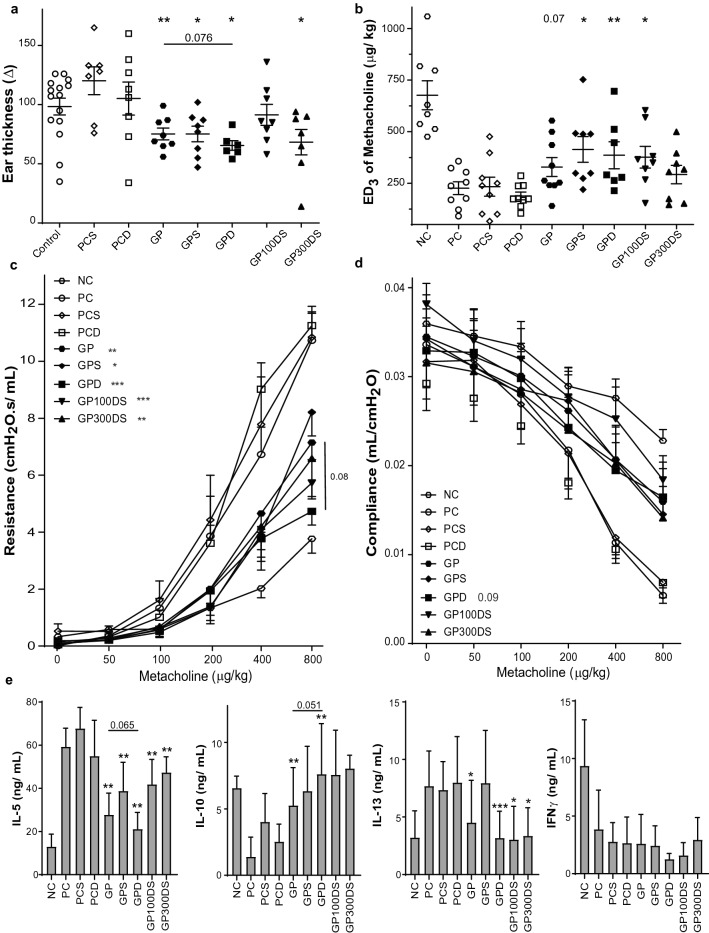
Clinical manifestations after VitD3-supplemented GP-SCIT. (**a**) Net ear thickness (mm) 2 h after GP injection performed after SCIT. Placebo-SCIT treated mice were plotted together as Controls (NC and PC). (**b**) Effective Dose (ED) of Methacholine, when the airway resistance reaches 3 cmH_2_O s/mL. (**c**) Airway hyperactivity (AHR) was plotted as Resistance (R in cmH_2_O s/mL) and as (**d**) Compliance (C in mL/cmH_2_O). (**e**) Net levels of IL-5, IL-10, IL-13, and IFNγ in restimulated lung cell suspensions. Mean ± SEM (n = 8). *NC* Negative Control, PBS challenged; *PC* Positive Control, GP challenged; *PCS* PC with 300 nmol SAINT; *PCD* PC with 300 ng VitD3 in SCIT; *GP* 300kSQ GP in SCIT; *GPS* 300kSQ + 300 nmol SAINT; *GPD* 300kSQ + 300 ng VitD3; *GP100DS* 300kSQ + 100 ng VitD3 + 300 nmol SAINT; *GP300DS* 300kSQ + 300 ng VitD3 + 300 nmol SAINT. *P < 0.05, **P < 0.01, ***P < 0.001 compared to their own matching controls, unless otherwise specified.

Next, we assessed whether the effect of SAINT mixed with VitD3-GP-SCIT improves the early-phase response to intradermal GP injections. Ear swelling was reduced in GP-SCIT groups as compared to their controls, and similar as previously; addition of VitD3 in GP-SCIT resulted in a trend towards suppression when compared to GP-SCIT (Fig. 5a). Addition of SAINT to the mixture did not further improve the swelling responses in the VitD3-GP-SCIT animals.

To measure effects of SAINT and VitD3-GP-SCIT mediated suppression of AHR, we measured airway resistance (R) and compliance (C) and calculated the ED_3_ values (R of 3 cmH_2_O s/mL) (Fig. 5b–d). The ED3 values were significantly increased compared to controls in all experimental groups except for the high-dose SAINT-VitD3-GP-SCIT group, however no net effect of VitD3 supplementation was found (Fig. 5b). In addition, in analyzing the dose–response curve of airway resistance, we observed a significant decrease in all GP-SCIT groups compared to the controls, and found a trend towards suppression in the VitD3-GP-SCIT group when compared to GP-SCIT mice (Fig. 5c). Nonetheless, addition of SAINT did not result in an increased suppression of AHR. For lung compliance, only the VitD3 supplemented GP-SCIT showed a significant improvement compared to the sham-treated positive controls (PCD), however, no effect of the use of SAINT was detected (Fig. 5d).

Restimulation of lung cell suspensions with allergen extract ex vivo to detect any allergen-induced cytokines revealed marked suppression of IL-5 and IL-13 production in all SCIT mice (Fig. 5e). Addition of VitD3 in GP-SCIT resulted in a trend towards suppression in IL-5 when compared to unsupplemented GP-SCIT mice. Furthermore, we observed increased IL-10 levels in these allergen recall cultures in all GP-SCIT treated groups, but due to high variability with the groups this was significant only for unsupplemented and VitD3-GP-SCIT groups, when compared to their matched controls. Addition of VitD3 did result in a trend towards increased IL-10 levels, when compared to GP-SCIT treated animals (Fig. 5e). Although inclusion of SAINT was unable to improve Th2 inflammatory status alone, VitD3 supplementation did result in augmented suppression of Th2 cytokine production ex vivo.

### Effects of SAINT on suppression of eosinophilic inflammation and cytokine responses

**Figure 6:**
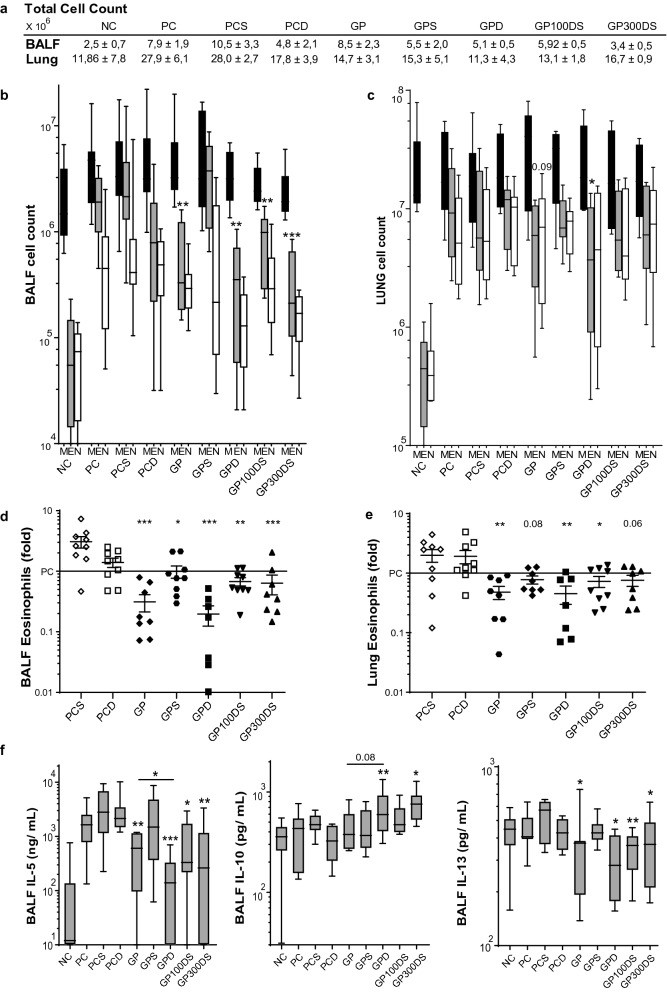
The eosinophilic and cytokine response after VitD3-GP-SCIT. (**a**) Total cell counts in BALF and Lung. (**b**) Differential cytospin cell counts in BALF and in (**c**) Lung. *M* mononuclear cells, *E* eosinophils, *N* neutrophils. (**b**, **c**) Box-and-whiskers plots (min–max). (**d**) BALF and (**e**) Lung eosinophils, both plotted as ratio of suppression (absolute EO/average PC EO; mean ± SEM). (**f**) Levels of IL-5, IL-10, and IL-13 in pg/mL measured in BALF; mean ± SEM (n = 8). *NC* Negative Control, PBS challenged; *PC* Positive Control, GP challenged; *PCS* PC with 300 nmol SAINT; *PCD* PC with 300 ng VitD3 in SCIT; *GP* 300kSQ GP in SCIT; *GPS* 300kSQ + 300 nmol SAINT; *GPD* 300kSQ + 300 ng VitD3; *GP100DS*, 300kSQ + 100 ng VitD3 + 300 nmol SAINT; *GP300DS* 300kSQ + 300 ng VitD3 + 300 nmol SAINT. *P < 0.05, **P < 0.01, ***P < 0.001 compared to their own matching controls, unless otherwise specified.

To assess effects of SAINT in VitD3-GP-SCIT on inflammation, we compared cell counts of eosinophilic granulocytes and levels of cytokines in lung tissue after GP-SCIT (Fig. 6a–c). We observed a marked suppression of eosinophil numbers in BAL fluid and lung tissue after GP-SCIT, irrespective of the use of VitD3 and SAINT (Fig. 6b,c). However amongst these striking results, we were unable to detect a net SAINT mediated effect on GP-SCIT, which was also evident when the data were presented as fold reduction in eosinophils of both GP-SCIT treated groups relative to their matched controls (Fig. 6d,e).

Finally, we also analyzed cytokine levels in BAL fluid and observed that levels of IL-5 and IL-13 were significantly affected by GP-SCIT, but we failed to detect additional suppression by VitD3 with or without SAINT (Fig. 6f). However, we were able to show a significant increase of IL-10 in the VitD3-GP-SCIT group compared to the matched positive controls, both in absence and presence of SAINT.

## Discussion

We previously reported the capacity of VitD3 to enhance efficacy of GP-SCIT in inducing a GP-spIgG2a response, suppressing eosinophils and enhancing IL-10 levels in lung tissue^21^. However, clinically relevant parameters such as airway hyperresponsiveness were not affected by VitD3 use. Therefore, in the current study, we analyzed the dose-dependent effects of VitD3 on GP-SCIT treatment, and we for the first time report a clear effect of high-dose VitD3 when compared to the unsupplemented GP-SCIT group: lower total- and GP-spIgE levels and an enhanced GP-spIgG1 response resulting in increased neutralizing activity, as well as suppression of ear swelling responses and airway resistance, Th2 cytokines, and eosinophils in BALF and lung tissue (Supplemental Table 1). Importantly, we report a clear correlation between dose of VitD3 during GP-SCIT and ED3 after allergen challenges, which reflects the responsiveness to methacholine in the lower part of the dose–response curve. These data identify airway resistance as one of the most sensitive parameters to increasing dosages of VitD3. Addition of the synthetic, bio-compatible lipid SAINT as carrier for the lipophilic VitD3, however, did not further enhance the VitD3-dependent suppression of allergic manifestations, even at suboptimal GP-dosages.

Previously, we showed that VitD3 enhanced effects of SCIT in an OVA-driven mouse model for allergic airway inflammation^25^. Herein, co-administration of 10 ng VitD3 with suboptimal OVA-SCIT enhanced suppression of AHR and eosinophilia, and increased levels of IL10 and TGF-β. More recently, we provided evidence that 10 ng VitD3 used as adjuvans in GP-SCIT and GP-SLIT augments induction of neutralizing antibodies, results in enhanced eosinophil-suppression and IL-10 production in lung tissue^11^. In the current study, we tested the dose dependent VitD3 effects in GP-SCIT, and show clear dose-dependent effects for some parameters only, while optimal suppression of the parameters of allergic airway inflammation by GP-SCIT are achieved at the highest dose of VitD3 (300 ng per injection). The difference in efficacy of VitD3 between the OVA and GP models (10 ng vs. 300 ng VitD3 per injection) most likely stems from the use of a purified protein (OVA), which has tolerogenic properties upon repeated administration^26^ versus a natural GP extract, which contains multiple allergens and may be immune-stimulatory. We feel that the SCIT model using the GP extract is a better translational model for the clinical practice where treatment depends on the use of allergen extracts or modifications thereof^23^.

In clinical studies, VitD3 insufficiency is thought to contribute to asthma^27^ and supplementation of VitD3 resulted in a reduction of the number of exacerbations in asthmatic patients^28^. Generally, its mechanism of action involves both directing the immune system towards a tolerogenic response, and enhancing epithelial barrier function^29,30^. Increased risk for persistent asthma in VitD deficient children may be associated with enhanced susceptibility to allergic sensitization^31^, while VitD supplementation provides clinical improvement in atopic disease^32^. Wolsk et al. found a reduction in the risk of recurrent wheeze and acute respiratory tract infections in early life after VitD3 supplementation during pregnancy^33^. In addition to baseline effects on asthma susceptibility, VitD3 might also improve the response to AIT. For instance, in allergic rhinitis patients, clinical effect of SCIT was more pronounced in patients whose VitD serum levels were sufficient during treatment^34^. In contrast, VitD supplementation in HDM-SCIT treated asthmatic children had no effect on symptom scores, spIgG1, nor regulatory cytokines IL-10 and TGF-β, although a slight increase in expression of FOXP3^+^ T cells was observed hinting its potential as AIT-adjuvants^16^.

Experimental mouse models of allergic airway disease have been used to further evaluate the effect of VitD3 levels on clinically relevant parameters^30^. Similar to the clinical studies, perinatal VitD deficiency in mice has immunomodulatory effects such as Th2 skewing and reduced numbers IL-10^+^ Tregs, both exaggerated upon HDM challenges^35^. Heine et al. showed that VitD deficiency in mice promotes allergic sensitization to OVA, while co-administration of 25(OH)D in OVA-SCIT reduced airway inflammation, Th2 cytokines in the lungs, and AHR after challenges^36^. Moreover, VitD supplemented Der p2-allergoid SCIT enhanced reduction of Th2 cytokines and eosinophilia and induced increased numbers of Tregs^37^. In our GP-SCIT mouse model, we report a similar suppression of Th2 activity by VitD3 supplementation. In contrast to our previous study using 10 ng VitD3, however, we did not observe induction of IL-10 in either lung tissue or in restimulated lung cells. Interestingly, we additionally observed suppression of a number of innate cytokines and chemokines in lung, including eotaxin (CCL11), GM-CSF, IL-33, and KC. The mechanism for this modulation of the lung innate immune response is unclear, but could involve enhanced induction of Treg cells after SCIT^38^, due to VitD3-dependent tolerogenic effects on the local APC. The suppression of the innate responses in lung-resident cells might be in line with enhanced Treg activity, although direct, systemic effects of the locally applied VitD3 cannot be excluded^28^.

Next, we tested whether use of SAINT in GP-SCIT would enhance tolerance induction after GP-SCIT injections by acting as a lipophilic carrier for VitD3. A SAINT-18 molecule contains two hydrophobic tails and one cationic pyridinium head group and builds complexes with the liposomal structures^20^. Similar as previous findings using carrier formulations like chitosan and PLGA-nano particles, SAINT-18 captures allergens and could induce an enhanced allergen uptake and presentation. For instance, Liu et al*.* reported beneficial effects of using chitosan as a chitosan-Der f nano-vaccine in a mouse model of intranasal AIT^39^. Also in a murine model, Saint-Lu et al*.* showed chitosan formulations to have mucoadhesive properties, induced enhanced uptake of OVA when applied sublingually and enhanced tolerance induction via lowered AHR, eosinophils, as well as specific Th2 responses^40^. More recently, PLGA-nano particles were used to enhance efficacy of SLIT in a murine model of allergic rhinitis^41^.

The crucial determinant in the treatment efficacy might be the particle size of the new formulations. The smaller the particles the better the antigen uptake and presentation, and classic liposomal formulations generally range between 50–250 nm in size, and are unstable below 50 nm due to the high curvature this requires for the lipids^42,43^. Moreover, smaller sized particles could possess adjuvant activity towards activation and maturation of DCs in a inducer of humoral and cellular immunity^44^. Our SAINT/GP formulations were prepared fresh and prepared in a standard sequence (WFI, SAINT-18, VitD3 and as a last component GP), resulting in a stable formulation with acceptable particle sizes (464–474 nm; 43.9 Vol%, data not shown). However, in pilot stability experiments, we did find that at higher dosages of VitD3, the mixture became more emulsified, and particle size would decrease to 78–80 nm (71.6 Vol%), possibly leading to more efficient phagocytosis by the APCs. However, we show that while VitD3 improves GP-SCIT, this effect was not enhanced by use of SAINT-18 as a carrier, arguing against an increased phagocytosis by the APCs.

Our study design also had some limitations, including the inclusion of VitD3 with the SCIT injections, which will result in local, but not systemic increases in VitD3 levels. Previously, it was found that VitD3-enriched chow (10,000 IU/kg) resulted in decreased AHR and airway inflammation compared to standard chow (2000 IU/kg)^45^. We used a diet containing 2900 IU/kg VitD3 in all groups, while VitD3 for SCIT supplementation was administered in the allergen injections, thereby selectively increasing local VitD3 levels, with the aim to induce a tolerogenic phenotype in the DCs responsible for allergen presentation. As we also see an effect on AHR and airway inflammation, similar to systemic VitD3 levels, we conclude that local effects are sufficient for the VitD3-mediated enhanced suppression of GP-SCIT.

Another limitation of our study is that not all VitD3-effects on GP-SCIT mediated suppression were observed in both experiments (dose-finding vs. use of SAINT as a carrier). To allow for an overall comparison between the two experiments, we have included a comparison of the experimental parameters between the two experiments in Supplemental Table 1. The VitD3 dose-finding experiment showed marked effects of VitD3 supplementation at the highest dose on most of the experimental parameters, whereas in the SAINT experiment, addition of VitD3 to GP-SCIT only had an effect on a subset of the experimental parameters, including the neutralizing antibody response, GP-induced ear swelling, and cytokine responses in lung tissue. The VitD3 effects on AHR and eosinophilic inflammation in GP-SCIT mice did not reach statistical significance in the SAINT experiment. In this respect, it is important to note that these two experiments were performed using different batches of *Phleum pratense* extract, which might explain some differences. Notwithstanding these subtle differences between experiments, we clearly show a reproducible effect of VitD3 on the efficacy of GP-SCIT.

In this study, VitD3 supplementation of GP-SCIT dose-dependently induced significantly enhanced suppression of spIgE, inflammation and hyperresponsiveness, while neutralizing capacity was improved and ESR were reduced. Addition of VitD3 further decreased Th2 cytokine responses and innate cytokines to allergens in lung tissue by GP-SCIT. In contrast, addition of synthetic lipid, SAINT to the GP-VitD3 mix had no therapeutic effect. These studies underscore the relevance of VitD3 as an adjuvant to improve clinical efficacy of SCIT treatment regimens.

## Supplementary information

Supplementary Information 1.

Supplementary Legend.
