# Supplementary material for: High dose vitamin D3 empowers effects of subcutaneous immunotherapy in a grass pollen-driven mouse model of asthma
Source: Sci Rep. 2020 Nov 30;10:20876. doi: 10.1038/s41598-020-77947-6 (PMC7705678; doi:10.1038/s41598-020-77947-6)
Supplement: Supplementary file 1 — Supplementary Information 1. [file 41598_2020_77947_MOESM1_ESM.pdf]

High dose vitamin D_3 empowers effects of subcutaneous immunotherapy in a grass pollen-driven mouse model of asthma

Laura Hesse MSc[1,2], N. van Ieperen[1,2], Arjen H. Petersen BSc[3], J.N.G. Oude Elberink[4], Antoon J.M. van Oosterhout[1,2], Martijn C. Nawijn[1,2*]

1: University of Groningen, University Medical Center Groningen, Department of Pathology & Medical Biology, Experimental Pulmonary and Inflammatory Research (EXPIRE), Groningen, The Netherlands.

2: Groningen Research Institute of Asthma and COPD (GRIAC), University of Groningen, University Medical Center Groningen, Groningen, The Netherlands.

3: University of Groningen, University Medical Center Groningen, Department of Pathology & Medical Biology, Medical Biology section, Groningen, The Netherlands.

4: University Medical Centre Groningen, Department of internal medicine, Division of Allergy, Groningen, The Netherlands.

* Corresponding author:

Dr. Ir. M.C. Nawijn, Assistant Professor

Experimental Pulmonary and Inflammatory Research (EXPIRE)

Department of Pathology and Medical Biology

Groningen Research Institute of Asthma and COPD (GRIAC)

University Medical Center Groningen (UMCG)

Internal postcode EA52

Hanzeplein 1

9713 GZ Groningen, The Netherlands

Telephone: +31 50 361 0998

FAX number: +31 50 361 9007

E-mail: m.c.nawijn@umcg.nl

S.Table 1: Overview of parameters of inflammation after GP SCIT with and without VitD to allow for direct comparison of both experiments

	SCIT			SAINT SCIT		
	**GP**	**GPD**	**P value**	**GP**	**GPD**	**P value**
Immunoglobulins	median (0ng VitD)	median (300ng VitD)	GP - GPD	median (0ng VitD)	median (300ng VitD)	GP - GPD
Post Total IgE (ng/mL)	45 x10^3	41 x10^3	*0,080*	20 x10^3	21 x10^3	
Post GP-spIgE (AU/mL)	70 x10^3	25 x10^3	*0,095*	7,6 x10^3	3,4 x10^3	
Post GP-spIgG1 (AU/mL)	42 x10^6	60 x10^6		20 x10^6	41 x10^6	*0,090*
Post GP-spIgG2a (AU/mL)	11 x10^3	3 x10^3		34 x10^3	66 x10^3	*0,090*
IgG1 Neutralizing activity after challenge (GP-spIgG1 / GP-spIgE Post)	6,6 x10^3	40 x10^3	*0,080*	4,3 x10^3	3,6 x10^3	
IgG2a Neutralizing activity after challenge (GP-spIgG2a / GP-spIgE Post)	2,2	1		16	4	
Fold induction of IgE after challenge (vs Median of PC or PCD)	2,62 (vs 187,6)	10,82 (vs 84,8)		8,98 (vs 235,1)	2,35 (vs 286,9)	*
AHR						
Fold induction of ED3	1,2	2,4	**	1,2	1,7	
Fold reduction Resistance at 400µg/kg MCh vs average of PC or PCD	0,83	0,35	*0,058*	0,78	0,42	
Fold reduction Compliance at 400µg/kg MCh vs average of PC or PCD	0,69	2,24	*0,102*	1,10	1,70	
EST						
Fold reduction EST after 2h	0,75	0,61	*0,090*	0,90	0,53	*0,076*
Inflammation						
Suppression of EO in BALF (ratio vs mean PC or PCD)	0,21	0,04	*	0,16	0,09	
Suppression of EO in Lung (ratio vs mean PC or PCD)	0,79	0,41	*0,090*	0,46	0,35	
Cytokines						
Cytokines in BALF				IL-5 and IL-13 ↓	IL-5 and IL-13 ↓ and IL-10 ↑	*
Cytokines in lung cells after GP stimulation *ex vivo*	NS	IL-5 and IL-13 ↓	*0,080*	IL-5 and IL-13 ↓ and IL-10 ↑	IL-5 and IL-13 ↓ and IL-10 ↑	*0,051*
Cytokines in lung tissue homogenates	GM-CSF suppression	IL-4, IL-5, IL-13, eotaxin, IL-33, GM-CSF, and KC ↓	*			

SFig 1

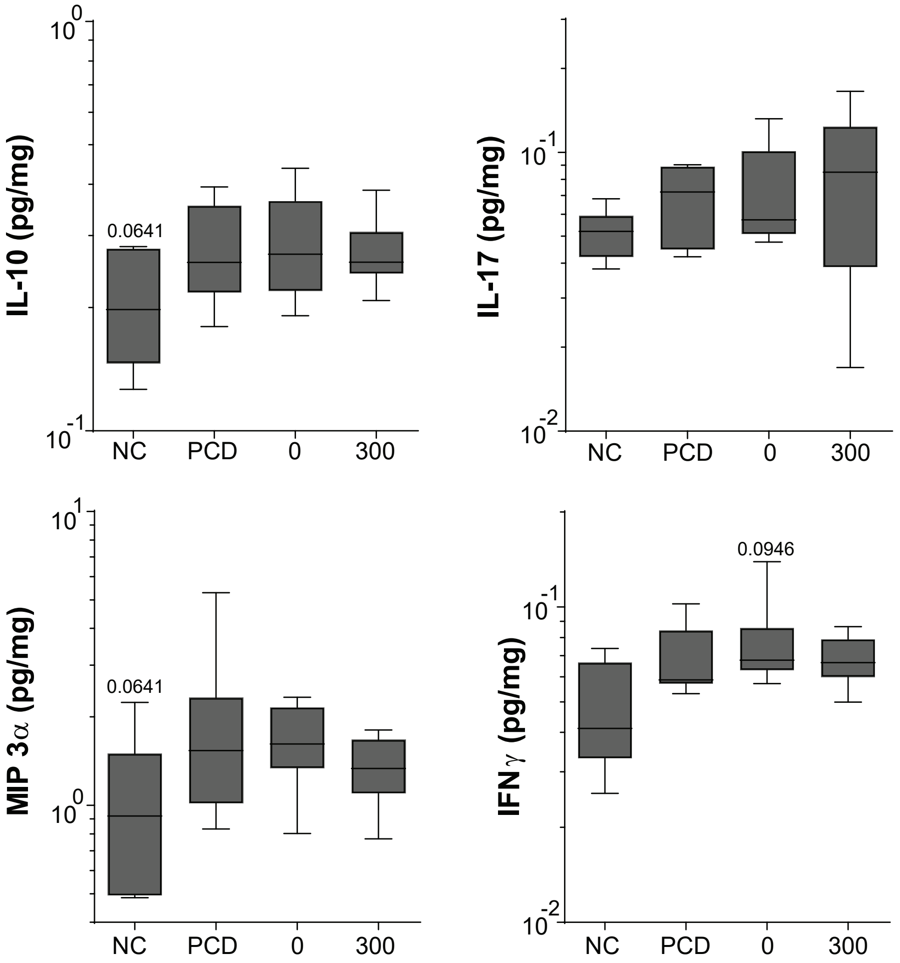
IL-10 (pg/mg)
IL-17 (pg/mg)
MIP 3α (pg/mg)
IFNγ (pg/mg)
0.0641
0.0641
0.0946
NC
PCD
0
300
10^{0}
10^{-1}
10^{-2}
10^{1}
