# Supplementary material for: High dose vitamin D3 empowers effects of subcutaneous immunotherapy in a grass pollen-driven mouse model of asthma
Source: Sci Rep. 2020 Nov 30;10:20876. doi: 10.1038/s41598-020-77947-6 (PMC7705678; doi:10.1038/s41598-020-77947-6)
Supplement: Supplementary file 2 — Supplementary Legend. [file 41598_2020_77947_MOESM2_ESM.docx]

**Supplemental Figure 1** Cytokine and chemokine responses after VitD3-GP-SCIT. Levels of IL-10, IL-17, MIP-3α and IFN-γ in pg/mg protein in lung tissue (mean ± SEM, n=8). NC: Negative Control, PBS challenged; PC: Positive Control, GP challenged; PCD: PC with VitD3 in SCIT (300ng); 0, 30, 100, and 300 groups all contain 300kSQ GP with 0, 30, 100, and 300 ng VitD3 respectively. *P<0.05, **P<0.01, ***P<0.001 compared to PC or PCD: unless otherwise specified.
